# Quantifying the relationship between physical performance and mental wellbeing in older adults: a field study

**DOI:** 10.3389/fragi.2025.1630343

**Published:** 2025-09-24

**Authors:** Róbert Járai, Erzsébet Stephens-Sarlós, Ferenc Ihász, Celal Bulgay, Ádám Balog, Anna Horváth-Pápai, Zoltán Alföldi, Eliza E. Tóth, Angéla Somogyi, Robert Podstawski, Attila Szabo

**Affiliations:** ^1^ Faculty of Health and Sport Sciences, Széchenyi István University, Győr, Hungary; ^2^ Faculty of Sports Science, Bingöl University, Bingöl, Türkiye; ^3^ Doctoral School of Health Sciences, Faculty of Health Sciences, University of Pécs, Pécs, Hungary; ^4^ Doctoral School of Psychology, Faculty of Education and Psychology, ELTE Eötvös Loránd University, Budapest, Hungary; ^5^ Department of Physiotherapy, University of Warmia and Mazury, Olsztyn, Poland

**Keywords:** aging, fitness, functionality, mental health, structural equation modeling

## Abstract

**Introduction:**

Although the relationship between functionality, as reflected in physical performance (PHP), and mental health in older adults has been researched, its strength remains unclear.

**Methods:**

This field study aimed to determine the strength of this relationship in adults aged 60 and above using seven PHP indices and six psychological measures. We individually tested 114 older adults. Objective measures included six PHP indices consisting of the Senior Test and handgrip strength. Subjective measures included resilience, wellbeing, happiness, perceived stress, hopelessness, and life satisfaction.

**Results:**

Structural equation modeling (SEM) revealed two latent constructs: PHP and mental wellbeing (MWB): robust fit (MLR): X^2^ (75) = 136.28, p < 0.001; CFI = 0.967; TLI = 0.960; RMSEA = 0.066 (90% CI [0.000, 0.128]); SRMR = 0.088. The latent partial correlation between PHP and MWB (adjusted for Age) was φ = 0.46, indicating ∼21% shared variance. The correlation between the two latent factors was moderate (r = 0.46), suggesting that other unassessed factors might account for the relationship.

**Discussion:**

Based on objective PHP and subjective MWB measures, these results suggest a modest connection, with the two latent constructs sharing ∼1/5 of their variances. Consequently, further research is needed to identify other factors affecting the studied relationship in older adults. These cross-sectional findings, suggesting a moderate association, should be interpreted with caution. Still, they support recommending physical activity as one component of broader, multi-domain strategies to support the wellbeing of older adults.

## Introduction

Good physical performance (PHP), necessary in everyday living, is maintained through engaging in active leisure activities, sports, exercise, and accomplishing daily energy-demanding physical activities (Caspersen et al., 1985). It plays a significant role in responding to unexpected challenges with sufficient energy and avoiding premature fatigue. PHP is closely associated with superior mental health (Firth et al., 2020), a relationship observed across various populations, including older adults (Cunningham et al., 2020). In this population, maintaining adequate PHP relies on regular physical activity (Aoyagi et al., 2009), that is connected with emotional resilience (Carriedo et al., 2020; Springfield et al., 2022), mental wellbeing (MWB; Carriedo et al., 2020; Gothe et al., 2019; Kim et al., 2016), optimism (Kim et al., 2016; Ryu et al., 2023), life satisfaction (An et al., 2020; Antunes et al., 2019; Kim et al., 2016), and happiness (An et al., 2020; Lin et al., 2020).

Despite these associations, many studies have focused on only a limited range of psychological indices and relied on self-reported questionnaires to measure PHP rather than objective functional fitness assessments. This limitation weakens the evidence for a robust connection between mental wellbeing (MWB), PHP, and its components in older adults. Kadariya et al. (2019) emphasized the need for studies using objective measures of PHP. Functional fitness, often assessed through body composition (e.g., body mass index [BMI]), strength, flexibility, coordination (agility and balance), and endurance, represents the cornerstone of exercise gerontology (Miotto et al., 1999; Roy et al., 2010). These elements are comprehensively measured by the Fullerton Functional Fitness Test (FFFT; Rikli and Jones, 1999; 2013), a validated tool supported by evidence of stability, reliability, and discriminant validity (Miotto et al., 1999).

The FFFT, also known as the “Senior Fitness Test” (Pepin et al., 2004), evaluates PHP in older adults by measuring upper and lower body strength, flexibility, coordination, and endurance through tasks such as walking, weightlifting, reaching, and balancing—activities integral to daily life. Healthcare professionals and fitness practitioners utilize the FFFT to identify areas of PHP that need improvement and to design personalized exercise programs. Although substantial research has explored the relationship between PHP and psychological wellbeing (Cunningham et al., 2020), few studies have examined the connection between PHP and a battery of MWB components. Understanding this relationship is vital in designing exercise programs that enhance PHP and MWB of older adults.

Recent studies suggest that various components of PHP may have distinct effects on MWB outcomes. For instance, Rajabpour et al. (2023) demonstrated that an 8-week, thrice-weekly fall prevention program improved coordination and MWB in older adults. Similarly, Gacek et al. (2023) reported a positive association between life-satisfaction coordination and endurance, as assessed by a 2-min step-in-place task. These findings underscore the importance of identifying the specific contributions of PHP components to MWB. Such insights can inform the development of targeted physical activity interventions that optimize MWB benefits by focusing on critical PHP elements.

Furthermore, a recent study investigating the relationship between PHP components and MWB in 39 older adults (Tóth et al., 2025) showed that body mass index (BMI), upper body strength, and upper body flexibility were the least significant predictors of MWB. In contrast, endurance, complex movement coordination, and lower body flexibility were the most significant. Mental resilience was strongly correlated with complex coordination and endurance. Endurance emerged as a predictor of optimism and satisfaction with life. Lower body flexibility was the most robust predictor of wellbeing and happiness. In contrast, lower body strength showed significant correlations with all PWB measures but was a weaker predictor in regression models. Complex movement coordination was also associated with life satisfaction and optimism. Overall, this work reveals a connection between PHP and MWB. However, Tóth et al. (2025) suggest that research with larger samples is needed to confirm these relationships. Indeed, apart from the relatively small sample size, this study did not include key negative indices of MWB, like perceived stress and hopelessness associated with depression (Assari and Lankarani, 2016) and suicide (Bickford et al., 2019) in older adults.

The present study aimed to address these gaps using objective measures of PHP to evaluate their impact on MWB. Grounded in Cognitive Behavioral Theory (CBT; Beck, 1979), which posits that thoughts, emotions, behaviors, and physical sensations are interconnected and essential to MWB, this research explored how PHP components contribute to MWB. For example, greater PHP may be associated with more positive thoughts and emotions. While flexibility and strength are essential, mobility, coordination, and endurance may play even more critical roles in self-assessed PHP and its influence on MWB. We hypothesized that a battery of seven objective measures of PHP indices would be closely associated with six subjective measures of MWB in older adults, with a substantial inverse contribution from stress and hopelessness.

Beyond prior work that relied mainly on self-reported activity or a narrow set of psychological indices, our study combines an objective, multi-domain PHP battery (FFFT plus handgrip) with a comprehensive panel of positive and negative mental-health indicators. Methodologically, we model these as two latent constructs in SEM, reducing measurement error and enabling a theory-driven test of the physical–psychological linkage in a real-world, care-home field setting. This design clarifies what portion of MWB is attributable to PHP in late life.

## Materials and methods

### Participants

We recruited participants from nine care homes for older adults after obtaining approval from the management. Eligibility criteria include being 60 years or older, able to communicate with researchers, and capable of standing and walking. Exclusion criteria included physical conditions such as occasional dizziness, cardiorespiratory disease, loss of balance, untreated hypertension, and diagnosed mental dysfunctions. Before data collection, medical clearance was obtained for all potential participants. Volunteers signed an informed consent form, consenting to participate and the anonymous publication of the results in a group-based setting. They also completed the General Data Protection Regulation (GDPR) data-handling form. While 114 older adults met the inclusion criteria and volunteered, many failed to attend either the objective (PHP) or subjective (MWB) tests, which were pre-scheduled at different times in the morning in a counterbalanced order. Since participants could withdraw consent at any time, we considered their non-attendance to reflect exercising this right.

Most participants were females (n = 87/114). Their mean age was 75.88 (±7.06) years, ranging from 62 to 94. Females were slightly younger (75.28 ± 7.16 years) than males (77.81 ± 6.47 years), but the difference was not statistically significant. We requested weight and height (to calculate BMI) because earlier research had shown that, despite BMI being part of functional fitness (Miotto et al., 1999; Roy et al., 2010), it is unrelated or negligibly related to MWB measures (Tóth et al., 2025). Finally, because recruitment relied on volunteers from care homes and professional networks, the sample may not be representative of the broader population of older adults.

### Ethics

We conducted the study with ethical approval (permission No. SZE/ETT-2/2024 [V. 6]) from the Scientific Advisory Board of Széchenyi István University. This work adhered to the ethical guidelines outlined in the British Psychological Society’s Code of Human Research Ethics (British Psychological Society, 2021). Additionally, the research adhered to the principles outlined in the Declaration of Helsinki (World Medical Association, 2013) regarding the use of human participants. All participants signed a consent form for participation and the anonymous publication of the research results.

### Subjective measures

*Resilience -* We assessed resilience using the 10-item Connor-Davidson Resilience Scale (CD-RISC-10; Connor and Davidson, 2003). This assessment involves a five-point Likert scale, where 0 indicates “not true at all,” one indicates “rarely true,” two indicates “sometimes true,” three indicates “often true,” and four indicates “true nearly all the time.” A sample item is “*I am able to adapt when changes occur*” (Item 1). The total score ranges from 0 to 40 points. A higher score reflects a higher level of resilience. The internal reliability (Cronbach’s alpha [α]) of the CD-RISC-10 is 0.80.

*Wellbeing Index -* The Wellbeing Index (WHO-5; World Health Organization, 1998; Topp et al., 2015) is a five-item questionnaire that evaluates subjective wellbeing over the past 2 weeks. Participants rate each item on a six-point Likert scale, ranging from 0 (not present) to 5 (constantly present). The scale assesses positive mood, vitality, and general interests, with example items such as “I have felt cheerful and in good spirits” (Item 1). Scores range from 0 to 25, with higher scores indicating greater wellbeing. Its internal consistency (Cronbach’s α) was reported at 0.86, ranging from 0.70 to 0.90 across 15 European nations (Cosma et al., 2022). However, the retrospective nature of the WHO-5 introduces potential memory bias, a noted limitation.

Subjective *Happiness Scale -* The Subjective Happiness Scale (SHS; Lyubomirsky and Lepper, 1999) is a concise four-item questionnaire measuring overall Happiness. Each item is rated on a seven-point Likert scale, from 1 (less happy) to 7 (much happier). Two items asked participants to compare their happiness to that of their peers. At the same time, the other two provide descriptions of happy and unhappy individuals, asking participants to rate how closely these descriptions match them. The SHS is popular for its simplicity and robust psychometric properties. Higher scores reflect greater happiness. The scale’s internal reliability (Cronbach’s α) ranges from 0.65 to 0.94 across 30 studies from more than 15 countries (Szabó, 2019).

Perceived *Stress Scale -* The Perceived Stress Scale-4 (PSS-4; Cohen et al., 1983) briefly measures perceived stress over the past month. It consists of four items rated on a five-point Likert scale from 0 (never) to 4 (very often), addressing feelings of unpredictability, uncontrollability, and being overwhelmed. Scores range from 0 to 16, with higher scores reflecting more perceived stress. The PSS-4 has demonstrated acceptable internal reliability (Cronbach’s α = 0.74) in a large sample from three Western European nations (Vallejo et al., 2018). Like the WHO-5, it collects retrospective data, but its longer timeframe (1 month versus 2 weeks) increases the risk of memory bias. Additionally, when completed, general psychological measures, including the PSS-4, may be influenced by current affective states (Szabo and Ábel, 2021).

*Beck Hopelessness Scale -* The Beck Hopelessness Scale-4 (BHS-4; Aish and Wasserman, 2001) assesses feelings of hopelessness and pessimism using four dichotomous (true/false) items. These items reflect negative attitudes about the future, self-worth, and the likelihood of positive changes. Total scores range from 0 to 4, with higher scores indicating more hopelessness. The BHS-4 is practical for its brevity and effectiveness in identifying hopelessness, a critical factor in assessing suicide risk. In a large-scale survey, the BHS-4 demonstrated strong internal reliability (Cronbach’s α = 0.85; Perczel Forintos et al., 2013).

*Satisfaction with Life Scale -* The Satisfaction with Life Scale (SWL; Diener et al., 1985) is a five-item self-report questionnaire assessing global cognitive judgments of life satisfaction. Each item is rated on a seven-point Likert scale, from 1 (strongly disagree) to 7 (strongly agree). Example items include “*In most ways, my life is close to my ideal*.” Scores range from five to 35, with higher scores reflecting greater life satisfaction. The SWL has demonstrated strong internal consistency (Cronbach’s α = 0.86) in a large sample of adults (Martos et al., 2014).

### Objective measures

*Fullerton Functional Fitness Test* - The FFFT assesses functional fitness, or PHP, on various mundane tasks (Jones and Rikli, 2000; Rikli and Jones, 1999; 2013). It includes the following measures:

1. Lower body strength (FFFT1) - 30s chair test, complete stand up and sit down (number of repetitions)
2. Upper body strength (FFFT2) – lifting 2 kg (women) or 3.5 kg (men) dumbbell while sitting on a chair and doing complete arm bends and stretches (number of repetitions in 30 s)
3. Upper body flexibility (FFFT3) - fingers touching behind the back (back scratch) ( ± cm)
4. Lower body flexibility (FFFT4)- forward bend from chair to extended leg (chair sit-and-reach) ( ± cm)
5. Complex coordination (agility, balance, and walking speed [FFFT5]) - standing up from a chair and avoiding a buoy 2.44 m away (8 feet) – recording duration in seconds
6. Endurance (physical effort [FFFT6]) - Two-minute walk-in-place test - records the number of whole steps completed in 2 minutes, raising each knee to the point halfway between the patella (kneecap) and iliac crest (top hip bone).

The FFFT is safe for both inactive and physically active older adults. Moreover, by examining everyday motor patterns, researchers can gain insight into the six PHP indices described above (Różańska-Kirschke et al., 2006).

*Handgrip Strength-* A CAMRY Model EH101 hand dynamometer (Zhongshan Camry Electronic Co., Ltd., Zhongshan, China) assessed handgrip strength. This ISO 9001-certified device, accredited by the Société Générale de Surveillance (SGS), offers a maximum capacity of 90 kg. It records and displays the peak grip strength during each measurement, providing reliable data for tracking maximum grip power. Researchers frequently use this instrument due to its proven accuracy and dependability (e.g., Bari et al., 2024). Calibration was performed according to the manufacturer’s specifications to ensure precision and consistency.

### Procedure

All tests were conducted at room temperature (23 C) on weekdays between 8:00 and 12:00 a.m. from early June 2024 to the end of September 2024. Data collection occurred individually in quiet rooms within the participants’ habitual environment. Objective (PHP) and subjective (MWB) tests were administered in a randomized order, with flexibility for rescheduling if participants were unable to attend their assigned testing time. For each participant, both tests were conducted on the same morning in separate rooms, one designated for objective testing and the other for subjective testing, with support from the care home management. While participants completed the questionnaires, a researcher was present but did not interact unless they had questions. After completing the shuffled questionnaires and demographic questions, participants were scheduled for the FFFT tests (or vice-versa). The researcher explained the FFFT and handgrip tasks twice—once verbally and once via video demonstration—before demonstrating the correct execution of each task. If participants were unsure, the researcher repeated the demonstration. The best performance from the two trials was recorded. Participants were debriefed and thanked for participating in the study after completing the objective and subjective tests.

## Results

Despite the significant difference in the gender ratio, we needed to examine whether we should control for potential gender differences. Therefore, we examined gender differences in all 11 dependent measures using Mann-Whitney nonparametric tests, supplemented with Monte Carlo simulations based on 10,000 sampled tables to enhance the accuracy and robustness of the calculated *p*-values. To account for Type I errors resulting from multiple tests, we employed the Holm-Bonferroni correction (Holm, 1979). The emerging results indicated that the genders differed only in upper body strength (FFFT2) and handgrip force (HGR), two natural physical differences between males and females. Specifically, the genders did not differ on any of the six psychological measures. Therefore, we did not control for gender in the subsequent statistical tests. The results of the Mann-Whitney tests, which served only as control measures, are illustrated in Table 1.

**TABLE 1:** Comparison of males (n = 27) and females (n = 87) on all dependent measures using Mann-Whitney U tests with Monte Carlo simulations based on 10,000 sampled tables with starting seed 1,502,173,562. After using the Holm-Bonferroni correction for multiple tests (to guard against Type I error), the two genders only differed in upper body strength (FFFT2) and handgrip force (HGR).

			FFFT1	FFFT2	FFFT3	FFFT4	FFFT5	FFFT6	HGR	RES	WHO	HAP	Stress	HPL	LSF
Mann-Whitney U			274.00	163.00	344.00	247.50	353.50	306.00	125.00	704.50	281.50	273.50	279.00	245.00	262.00
Wilcoxon W			1054.00	1024.00	534.00	437.50	543.50	1167.00	986.00	3554.50	347.50	1651.50	1710.00	311.00	1640.00
*Z*			−1.60	−3.61	−0.73	−2.15	−0.57	−1.33	−4.20	−0.74	−0.08	−0.23	−0.22	−0.82	−0.44
Asymp. Sig. (2-tailed, *p*)			0.11	<0.01	0.47	0.03	0.57	0.18	<0.01	0.46	0.93	0.82	0.82	0.41	0.66
Holm-Bonferroni corrected *p*			1.00	0.01*	1.00	0.39	1.00	1.00	<0.01*	1.00	1.00	1.00	1.00	1.00	1.00
Monte Carlo Sig	Sig		0.114^b^	<0.001^b^	0.479^b^	0.035^b^	0.575^b^	0.183^b^	0.000^b^	0.471^b^	0.938^b^	0.827^b^	0.827^b^	0.447^b^	0.671^b^
	99% Confidence Interval	LP	0.11	0.00	0.47	0.03	0.56	0.17	0.00	0.46	0.93	0.82	0.82	0.43	0.66
		UP	0.12	0.00	0.49	0.04	0.59	0.19	0.00	0.48	0.94	0.84	0.84	0.46	0.68

* Statistically significant difference; FFFT1-FFFT6 = Fullerton Functional Fitness Test components 1 to 6; HGR, handgrip strength; LSF, life satisfaction; RES, resilience; WHO, wellbeing; HAP, happiness; Stress = perceived stress; HPL, hopelessness.

b = Monte Carlo probability

For the primary test, we employed structural equation modeling (SEM) analysis using the R software version 4.4.1 (R Core Team, 2024) with the lavaan package version 0.6.19 (Rosseel, 2012) to examine the hypothesized relationship between MWB and functional fitness. We operationalized MWB as a latent construct measured by life satisfaction (LSF), wellbeing (WHO), happiness (HAP), resilience (RES), stress, and hopelessness (HPL). We defined functional fitness as another latent construct based on Fullerton Functional Fitness tasks (FFFT1-FFFT6) and a left-right average handgrip (HGR) strength test. We also included age as a control variable.

Before estimating structural relations, we specified a two-factor confirmatory measurement model (CFA) with MWB indicated by RES, WHO-5, SHS (HAP), PSS-4 (Stress), HPL, and LSF, and PHP indicated by the six FFFT tasks and handgrip (HGR). Indicators were constrained to load only on their intended factor; the latent factors were allowed to covary; no cross-loadings were permitted. Time-based FFFT tasks were coded such that higher times reflect poorer performance; thus, negative loadings on PHP are expected and were not reversed. Models were estimated with ML and FIML for missing data; model fit was evaluated using χ^2^/df, CFI/TLI, RMSEA (90% CI), and SRMR as recommended. We specified a two-factor CFA with latent variables for PHP and MWB. Age was modeled as a covariate of both factors (Age → PHP; Age → MWB), and we estimated the latent residual correlation (PHP ↔ MWB), yielding the association net of Age. Missing data were handled using FIML with robust maximum likelihood (MLR; see Supplement S2 for details and MAR checks).

Our structural equation model (Model 1) included MWB as one latent variable, with six subjective measures, and functional fitness as the other, with seven observed measures, and age as a control variable. We followed Hu and Bentler (1999) for the SEM model fit criteria, who recommended evaluating model fit using multiple indices. Specifically, these criteria included the Comparative Fit Index (CFI) and Tucker-Lewis Index (TLI) values greater than or equal to 0.95, a Root Mean Square Error of Approximation (RMSEA) value less than or equal to 0.06, and a Standardized Root Mean Square Residual (SRMR) value less than or equal to 0.08. We used these thresholds as guidelines and evaluated the fit holistically, emphasizing convergence across indices rather than adhering to strict cutoffs.

**FIGURE 1:**
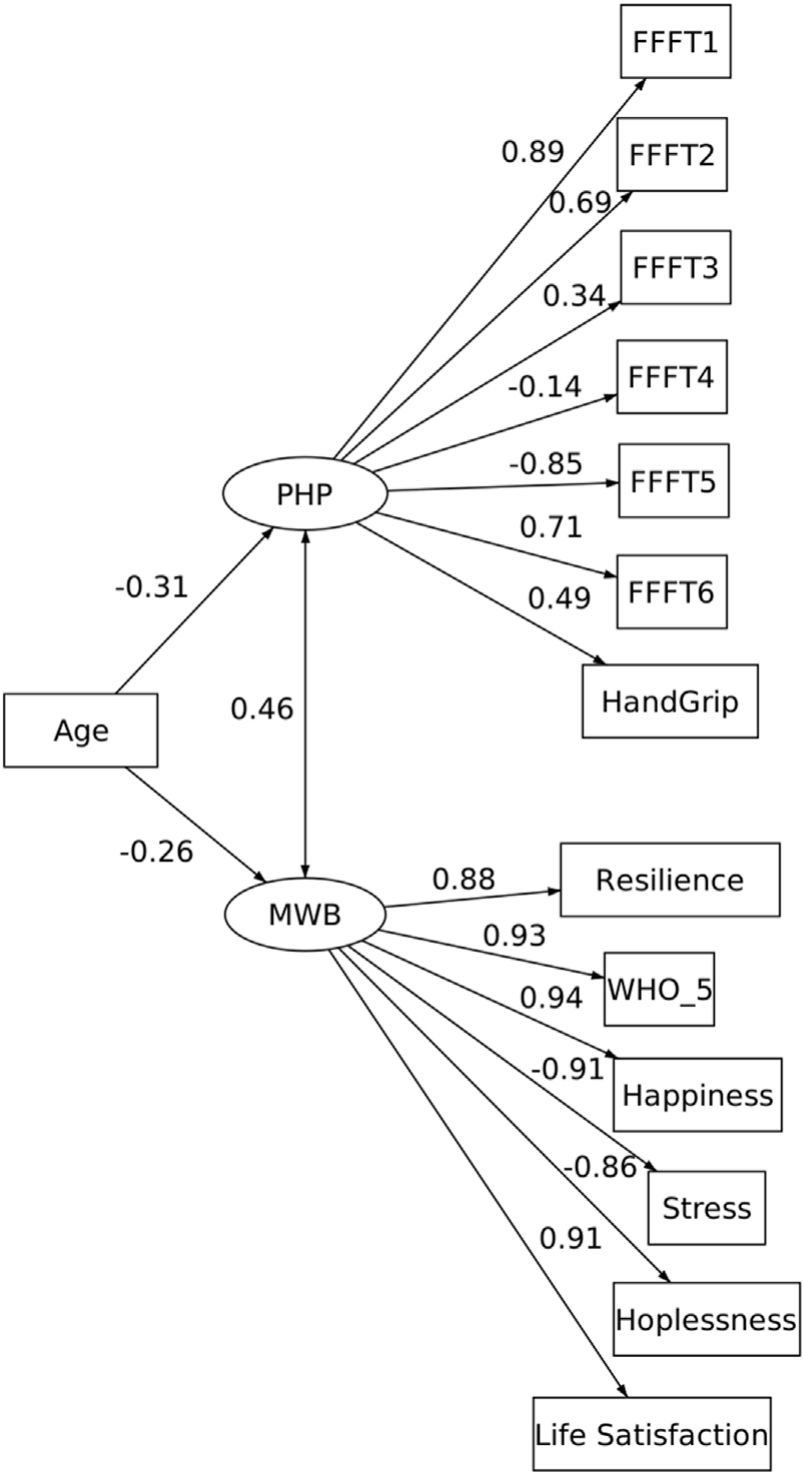
Structural equation model (SEM) of physical performance (PHP) and mental wellbeing (MWB) in older adults. PHP was modeled as a latent factor indicated by seven objective measures from the Fullerton Functional Fitness Test (FFFT1–6) and handgrip strength, while MWB was modeled as a latent factor indicated by six subjective measures (resilience, WHO-5 wellbeing, happiness, perceived stress, hopelessness, and life satisfaction). Standardized factor loadings and path coefficients are displayed. Age was included as a covariate of both latent factors, with an age-adjusted latent correlation estimated between PHP and MWB.

Overall model fit was good (Model 1): χ^2^ (75) = 136.280, p < 0.001, χ^2^/df = 1.817, RMSEA = 0.066 (90% CI [0.000, 0.128]), p = 0.507, CFI = 0.967, and TLI = 0.960. Given the small N and degrees of freedom, we interpret RMSEA cautiously and base conclusions on the convergence of multiple indices rather than any single cutoff. The standardized covariance coefficient between PHP and MWB, being latent variables, revealed a statistically significant, medium-strength, positive relationship: φ = 0.461; *p* < 0.001. In the measurement portion of the model, MWB loadings were strong (0.86–0.94, all p < 0.001), while PHP loadings ranged from −0.85 to 0.89 (|p| < 0.05 for all except FFFT4), consistent with the expected negative sign for time-based tasks. Table 2 shows the other variances and covariances in the model, while Figure 1 shows the standardized path coefficients.

**TABLE 2:** Standardized path coefficients for the physical performance and mental wellbeing model.

Outcome	Predictor	est.std	SE	*z*	*p*	CI lower	CI upper
PHP	FFFT1	0.892	0.036	24.462	0.000	0.820	0.963
PHP	FFFT2	0.687	0.136	5.070	0.000	0.422	0.953
PHP	FFFT3	0.344	0.150	2.301	0.021	0.051	0.638
PHP	FFFT4	−0.139	0.223	−0.626	0.531	−0.576	0.297
PHP	FFFT5	−0.850	0.058	−14.697	0.000	−0.963	−0.737
PHP	FFFT6	0.712	0.082	8.712	0.000	0.552	0.872
PHP	HGR	0.487	0.114	4.263	0.000	0.263	0.711
MWB	RES	0.875	0.041	21.405	0.000	0.795	0.955
MWB	WHO	0.932	0.020	47.791	0.000	0.894	0.970
MWB	HAP	0.941	0.018	52.347	0.000	0.906	0.977
MWB	Stress	−0.913	0.023	−40.302	0.000	−0.957	−0.868
MWB	HPL	−0.859	0.033	−25.775	0.000	−0.924	−0.793
MWB	LSF	0.905	0.029	31.015	0.000	0.848	0.963
PHP	MWB	0.461	0.117	3.941	0.000	0.232	0.691
PHP	Age	−0.313	0.137	−2.290	0.022	−0.581	−0.045
MWB	Age	−0.263	0.092	−2.861	0.004	−0.443	−0.083

predictor = predictor variable; outcome = outcome variable; est. std = standardized estimate; CI, confidence interval; FFFT1-FFFT6 = Fullerton Functional Fitness Test components 1 to 6; HGR, handgrip strength; LSF, life satisfaction; RES, resilience; WHO, wellbeing; HAP, happiness; Stress = perceived stress; HPL, hopelessness; MWB, mental wellbeing; PHP, physical performance.

## Discussion

The present study’s main contribution lies in *quantifying* the long-known and repeatedly demonstrated, albeit heterogeneously, association between physical and mental health in older adults. Indeed, in this study, PHP measures included only six indices of functionality and handgrip strength, both of which are strongly associated with MWB (Taekema et al., 2010; Tóth et al., 2025). By integrating objective functional indices with six PHP measures in a latent-variable SEM, we show that the physical–mental link is moderate rather than large, providing a quantitative upper bound (≈one-fifth shared variance) for older adults. This refines theory by tempering broad claims that ‘fitness equals wellbeing’ and points to additional psychological and social mechanisms that are likely to account for the majority of variance.

The results indicate a moderate relationship between PHP and MWB. The MWB factor was highly influenced by the six subjective measures (e.g., RES, WHO, HAP, Stress, HPL, LSF), as demonstrated by the high beta values (refer to Figure 1) ranging from 0.86 to 0.94, and exhibited a strong internal consistency. These high beta values indicate that the subjective dependent variables are strong predictors of MWB, suggesting that MWB is well-defined and captures a robust underlying construct. Thus, MWB explains a large portion of the variance in its corresponding observed variables, signaling that its construct is highly relevant to the study’s context and conceptualization of subjective indices as MWB. Hence, future studies could confidently use such a battery of variables.

The second factor, PHP, comprises seven observed variables with beta values ranging from 0.14 to 0.89, reflecting a varied strength of influence across its indicators. The higher beta values (e.g., 0.89) highlight strong predictors of the latent factor, while lower values (e.g., 0.14) suggest a weaker contribution. Notably, the strongest associations with PHP were observed in FFFT1, FFFT2, FFFT5, and FFFT6, corresponding to indices of lower and upper body strength, complex coordination, and endurance, respectively. These findings align with previous research identifying these factors as key predictors of functionality in older adults (Jones and Rikli, 2000; Pepin et al., 2004; Rikli and Jones, 1999; 2013).

Additionally, the results support using the modified FFFT, which includes handgrip strength (Migaj et al., 2022), as evidenced by the beta value of 0.49 (Figure 1), indicating a moderate-to-strong relationship. This result suggests that incorporating handgrip strength into the FFFT framework provides a more comprehensive functional fitness assessment. Therefore, future studies employing the modified FFFT may gather a more accurate and holistic evaluation of an individual’s functional fitness.

The standardized covariance of 0.46 between PHP and MWB indicates a moderate positive relationship between the objective physical and subjective psychological measures. This finding suggests that while PHP and MWB are distinct, they are still meaningfully related, sharing 21.25% of the variance, with changes in one being associated with changes in the other. However, considering the PHP and MWB in the current study only involved seven and six determinants of the full spectrum of physical functioning and mental health, the 21.25% shared variance could be considered meaningful. This moderate covariance underscores the idea that the constructs share some overlap but retain uniqueness. Our results align with an earlier work, which shows that self-assessed physical and mental health independently predict functional decline in older adults (Lee, 2000). They also expand Lee’s results because, in this study, the physical indices were objective, not subjective. Regrettably, we did not assess the subjective perception of physical functioning, which would allow us to calculate the correlation between the two.

Why might the PHP–MWB association be only moderate? Beyond measurement coverage (our model captures a subset of physical and psychological domains), unmeasured psychosocial resources (e.g., social support, perceived control/self-efficacy), lifestyle factors (such as sleep regularity and daily activity patterns), and health comorbidities can independently shape MWB, thereby diluting the bivariate link with functional performance. In addition, state–trait variability in mood and stress at assessment can attenuate cross-sectional associations even when the underlying relationship is meaningful. These considerations align with recent systematic reviews and meta-analyses, which typically report small to moderate associations under controlled design conditions (Noetel et al., 2024; White et al., 2024). In addition, observational and meta-analytic evidence linking functional strength with depressive symptoms in mid-to late life commonly yields inverse but non-large effects, consistent with our pattern (Zasadzka et al., 2021; Sun and Liu, 2024).

Psychosocial pathways, such as social connectedness and loneliness, may also play a role. Functional status and activity facilitate social participation, which is associated with better mental-health outcomes, whereas chronic loneliness is a risk factor (Hajek et al., 2024; White et al., 2024). Motivational–cognitive factors, particularly self-efficacy, may partly mediate the function → mental health pathway, as physical activity and self-efficacy are moderately and bidirectionally related in older adults (Xie et al., 2025). Lifestyle regulators such as sleep and diet also matter; sleep disturbance is closely linked to late-life depression, while Mediterranean-style diet patterns predict fewer depressive symptoms (Irwin et al., 2022; Wang et al., 2024). Finally, biological pathways, such as frailty, inflammation, and structural brain changes, may underlie part of the association, with physical activity reducing depressive risk partly through anti-inflammatory and neurotrophic processes (Jiang et al., 2024; Noetel et al., 2024).

While age was not directly tested as part of the hypothesis, it was included as a control measure and demonstrated standardized covariances of 0.31 and 0.26 with PHP and MWB, respectively (Figure 1). These moderate relationships suggest that age has some influence on both PHP and MWB, yet the effect is not particularly strong. The relatively modest covariances imply that other factors likely play a more significant role in explaining the variance in PHP and MWB. Nevertheless, the inclusion of age in the model underpins its expected relevance as a direct or indirect contributor to the constructs under investigation.

Finally, we acknowledge that the sample size (N = 114) was modest and participants were disproportionately female (76%) and recruited exclusively from care homes. These features limit the generalizability of our findings to the broader population of older adults, particularly community-dwelling men. At the same time, the gender imbalance reflects the demographic reality that women substantially outnumber men in nursing and care homes in later life, which makes recruitment of balanced samples in this setting challenging. Moreover, testing frail, older individuals who are both willing and medically cleared to participate is inherently difficult, which further constrains sample representativeness. Therefore, our findings should be interpreted with caution and viewed as specific to relatively healthy, care-home–dwelling older adults, while replication in more diverse and community-based samples remains necessary.

In summary, the results suggest that PHP and MWB are distinct but related, with several observed variables driving their measurement. The role of age is moderate, suggesting that other factors not captured in the model may contribute more strongly to the observed outcomes. Future research should investigate additional mediators and moderators, including social, psychological, lifestyle, and biological pathways, to enhance the understanding of these relationships. In doing so, particular attention should be paid to variables that vary by age group or other demographic characteristics, as well as to practical applications such as group-based functional training, self-efficacy building, sleep interventions, and dietary guidance (Irwin et al., 2022; Wang et al., 2024; Soong et al., 2025; Xie et al., 2025).

### Theoretical implications

The results of this study highlight a moderate positive relationship between PHP and MWB, suggesting that improvements in functional fitness are associated with enhanced mental wellbeing, consistent with the principles of Cognitive Behavioral Theory (CBT). The CBT posits that physical activity linked to functionality can directly and indirectly influence cognitive appraisals, emotions, and behavior. This interplay provides a framework for understanding the observed connection between PHP and MWB in older adults.

Conceptually, improvements in PHP may indirectly enhance MWB by increasing self-efficacy and autonomy, as well as expanding social participation opportunities; conversely, low MWB can suppress activity, suggesting reciprocal influences. We therefore posit a serial pathways model (PHP → self-efficacy/social engagement → MWB; to be tested), moderated by factors such as comorbidity burden and economic resources, which can be tested in longitudinal or intervention designs.

### Practical implications

The findings suggest that interventions grounded in CBT principles could simultaneously target physical and psychological dimensions. Enhancing physical functionality through structured exercise or functional training programs can augment cognitive appraisals and emotional regulation, while also improving mental wellbeing. Moreover, psychological interventions could reinforce positive beliefs about physical activity, encouraging older adults to engage in behaviors that enhance both physical and mental health. Future research should investigate potential mediators, such as self-efficacy, financial status, or social support, to gain a deeper understanding of the mechanisms underlying this relationship.

However, these practical implications should be interpreted with consideration for the study’s limited sample representativeness. Because our participants were largely female residents of care homes, the findings may not fully extend to community-dwelling men or to older adults with different living arrangements. Thus, while structured physical activity combined with psychosocial support remains a promising strategy to enhance wellbeing, practitioners and policymakers should exercise caution in generalizing our recommendations to populations with different demographic characteristics. Future intervention studies would benefit from tailoring programs to the needs of men and those living independently in the community.

In future research, interventions should combine PHP training (such as lower-body strength, balance, coordination, and endurance) with structured peer interaction to foster social support and goal setting based on mastery, thereby enhancing self-efficacy. Brief CBT-based elements (self-monitoring, realistic reframing, problem-solving) can be incorporated to help translate physical improvements into psychological benefits. Services should also screen for high stress or hopelessness and provide targeted psychosocial support to those individuals to maximize MWB impact.

Overall, it is important to note that the observed connection between physical performance and mental wellbeing was moderate, accounting for only about one-fifth of the variance. Therefore, physical activity interventions should be considered as one part of broader, multidomain strategies that also address social, psychological, and lifestyle factors to optimize wellbeing outcomes in older adults.

#### Limitations

The study has limitations that should be considered in the generalizability of the findings. First, the older adults studied were volunteers who had received medical clearance for physical and mental tests. Hence, they represent a relatively healthy older age group. In addition, because participation was voluntary, the sample may also represent more motivated residents compared with the general care home population. This selection bias could have led to somewhat inflated estimates of functional performance or wellbeing; therefore, the findings should be interpreted cautiously. Second, many missing data were compensated for with statistical methods in the data analyses. Third, the average of the two trials and general feelings on the testing day could have influenced the measured indices. Fourth, despite the suitable model fit, our measures, which mirrored PHP and MWB, were only part of the spectrum representing these concepts.

Furthermore, our results should be interpreted with caution, given the small sample size, the high proportion of female participants, and the recruitment of volunteers from care homes. These factors may favor healthier and more motivated residents, potentially inflating or attenuating the observed links between functional fitness and mental wellbeing, which could limit the generalizability of the findings to men, community-dwelling older adults, or other settings. Future work should utilize probability-based or registry sampling, include community samples, and predefine sex- and setting-stratified analyses to test robustness.

Finally, fit should be interpreted holistically: with CFI/TLI ≥0.96 alongside RMSEA ≈0.066, the overall evidence supports acceptable fit, acknowledging that RMSEA can be upwardly biased in smaller samples and lower-df models. We therefore emphasize the pattern of indices and the consistency of factor loadings when judging robustness.

## Conclusion

This study reinforces the significant yet nuanced relationship between functional physical performance (PHP) and mental wellbeing (MWB) in older adults, demonstrating that both constructs are distinct yet moderately correlated. Key observed variables, including lower body strength, flexibility, coordination, and upper body strength, underscore PHP as a vital part of functional fitness, while MWB represents strong psychological health indicators. The findings align well with the Cognitive Behavioral Theory, emphasizing the interplay between physical functionality and cognitive-emotional processes, suggesting that interventions addressing both dimensions may yield substantial benefits for older adults. Given the modest size of the association, physical performance should be recognized as one of several contributors to mental wellbeing in late life, alongside psychosocial, lifestyle, and health-related determinants. Future research should investigate additional mediators to deepen the understanding of these relationships and fine-tune or optimize interventions for enhancing holistic wellbeing in older.

## Data Availability

The datasets presented in this study can be found in online repositories. The names of the repository/repositories and accession number(s) can be found below: https://data.mendeley.com/datasets/8c9dgjjswk/1.
