# Supplementary material for: Quantifying the relationship between physical performance and mental wellbeing in older adults: a field study
Source: Front Aging. 2025 Sep 24;6:1630343. doi: 10.3389/fragi.2025.1630343 (PMC12504508; doi:10.3389/fragi.2025.1630343)
Supplement: Supplementary file 1 [file DataSheet1.pdf]

# Supplement for the Reviewer: SEM Estimation, Missing Data, and Parsimony Checks

## S1. Estimation framework and fit reporting

Analyses were conducted in R 4.4.1 (lavaan 0.6-19). Missing data were handled using Full Information Maximum Likelihood (FIML) with the robust maximum likelihood estimator (MLR). Robust (Yuan–Bentler) indices are reported. The primary two-factor model (MWB, PHP) specifies Age as a covariate of both factors (Age → PHP; Age → MWB) and estimates the Age-adjusted latent residual correlation (PHP ↔ MWB). Robust fit: CFI = .967; TLI = .960; RMSEA = .066 (90% CI [.000, .128]); SRMR = .088. The Age-adjusted latent correlation was $\varphi$ = .46 (≈21% shared variance). Multivariate normality was supported by Mardia's test (skew p = .188; kurtosis p = .392).

## S2. Extent and patterning of missing data

Sample size N = 114; complete cases = 27 (23.7%); distinct missing-data patterns = 10.

Per-variable missingness (SEM variables) ranged from 0.0% to 49.1% (Table S1).

**Table S1. Missingness by variable (N = 114)**

| Variable | Missing_n | Missing_% |
|---|---|---|
| Age | 0 | 0.0 |
| FFFT1 | 56 | 49.1 |
| FFFT2 | 54 | 47.4 |
| FFFT3 | 54 | 47.4 |
| FFFT4 | 55 | 48.2 |
| FFFT5 | 54 | 47.4 |
| FFFT6 | 54 | 47.4 |
| Resilience | 18 | 15.8 |
| WHO | 51 | 44.7 |
| Happiness | 51 | 44.7 |
| Stress | 50 | 43.9 |
| Hoplessness | 51 | 44.7 |

| | | |
|---|---|---|
| LifeSatisf | 51 | 44.7 |
| HandGrip_Left_and_Right | 54 | 47.4 |

**S2.2 Blockwise patterns (counts)**

• All physical measures missing (FFFT1–6, Handgrip): 54/114 (47.4%).

• All well-being indicators missing (Resilience, WHO-5, Happiness, Stress, Hopelessness, Life satisfaction): 17/114 (14.9%).

• Both blocks entirely missing (only Age observed): 11/114 (9.6%).

• Physical block missing while all six MWB indicators present: 34/114 (29.8%).

**S2.3 MAR plausibility checks (Age contrasts)**

To evaluate the plausibility of Missing At Random (MAR), we compared Age between "missing" vs. "observed" groups for each variable (Table S2). Standardized mean differences (Cohen's d) were generally small (median $|d| \approx 77.08$, maximum $|d| \approx 77.33$), suggesting that missingness is not strongly associated with Age.

**Table S2. Age comparison (missing vs. observed) by variable**

| Variable | Age_mean_missing | Age_mean_observed | Age_SMD_d |
|---|---|---|---|
| FFFT1 | 74.38 | 77.33 | 0.43 |
| FFFT2 | 74.48 | 77.13 | 0.38 |
| FFFT3 | 74.48 | 77.13 | 0.38 |
| FFFT4 | 74.58 | 77.08 | 0.36 |
| FFFT5 | 74.48 | 77.13 | 0.38 |
| FFFT6 | 74.48 | 77.13 | 0.38 |
| HandGrip_Left_and_Right | 74.48 | 77.13 | 0.38 |
| Resilience | 73.00 | 76.42 | 0.49 |
| WHO | 76.69 | 75.22 | -0.21 |
| Happiness | 76.69 | 75.22 | -0.21 |
| Stress | 76.86 | 75.11 | -0.25 |
| Hoplessness | 76.69 | 75.22 | -0.21 |

| | | | |
|---|---|---|---|
| LifeSatisf | 76.69 | 75.22 | -0.21 |

**S3. Model parsimony, sample size, and sensitivity analyses**

Although the primary model includes 13 indicators plus a covariate with N = 114, two factors support adequacy: (i) strong loadings/communalities (MWB ≈ .88–.94; several PHP ≥ .69) and (ii) simple structure (two factors, no cross-loadings). Methodological work (e.g., MacCallum, Browne, & Sugawara, 1996; Kline, 2016; Wolf, Harrington, Clark, & Miller, 2013) indicates that such models can be satisfactorily estimated around N ≈ 100–150 when loadings are strong. Sensitivity checks (removing the weakest PHP indicator or retaining only the strongest PHP indicators) yielded similar latent correlations and an acceptable robust fit, supporting the robustness of conclusions.

**S4. Reproducibility notes**

Software: R 4.4.1; lavaan 0.6-19. Estimation: MLR with FIML (missing = "fiml", estimator = "MLR"). Normality: Mardia (skew p = .188; kurtosis p = .392). Minimal code to reproduce the models is available upon request.

**References**


Allison, P. D. (2003). Missing data techniques for structural equation modeling. Journal of Abnormal Psychology, 112(4), 545–557. https://doi.org/10.1037/0021-843X.112.4.545

Kline, R. B. (2016). Principles and practice of structural equation modeling (4th ed.). Guilford Press.

MacCallum, R. C., Browne, M. W., & Sugawara, H. M. (1996). Power analysis and determination of sample size for covariance structure modeling. Psychological Methods, 1(2), 130–149. https://doi.org/10.1037/1082-989X.1.2.130

Wolf, E. J., Harrington, K. M., Clark, S. L., & Miller, M. W. (2013). Sample Size Requirements for Structural Equation Models. Educational and Psychological Measurement, 76(6), 913–934. https://doi.org/10.1177/0013164413495237


**S3b. Model fit and key path (robust indices)**

| Model | Robust CFI | Robust TLI | Robust RMSEA | SRMR | β PHP→MWB |
|---|---|---|---|---|---|
| Primary (13 inds) | .967 | .960 | .066 | .088 | .469 |
| Reduced PHP (drop FFFT4) | 1.000 | 1.007 | 0.000 | .077 | .467 |

| | | | | | |
|---|---|---|---|---|---|
| Parsimonious PHP (FFFT1,2,6,HGR) | .976 | .969 | .070 | .056 | .466 |

Notes: Robust TLI can exceed 1.00 with scaled corrections; values are reported as returned by lavaan. In both sensitivity models, the PHP → MWB path remained positive and statistically significant with a magnitude similar to the primary model.

## S4. Reproducibility notes and code

Software: R 4.4.1; lavaan 0.6-19. Estimation: MLR with FIML (missing = 'fiml', estimator = 'MLR'). Below are minimal code snippets to reproduce the primary and sensitivity models.

```
model_primary <- '
  MWB =~ RES + WHO + HAP + Stress + HPL + LSF
  PHP =~ FFFT1 + FFFT2 + FFFT3 + FFFT4 + FFFT5 + FFFT6 + HGR
  MWB ~ PHP + Age
  PHP ~ Age
'
fit <- sem(model_primary, data = data, missing = "fiml", estimator = "MLR")
summary(fit, fit.measures = TRUE, standardized = TRUE)

model_drop4 <- '
  MWB =~ RES + WHO + HAP + Stress + HPL + LSF
  PHP =~ FFFT1 + FFFT2 + FFFT3 + FFFT5 + FFFT6 + HGR
  MWB ~ PHP + Age
  PHP ~ Age
'
fit_drop4 <- sem(model_drop4, data = data, missing = "fiml", estimator = "MLR")
summary(fit_drop4, fit.measures = TRUE, standardized = TRUE)

model_pars <- '
  MWB =~ RES + WHO + HAP + Stress + HPL + LSF
  PHP =~ FFFT1 + FFFT2 + FFFT6 + HGR
  MWB ~ PHP + Age
  PHP ~ Age
'
fit_pars <- sem(model_pars, data = data, missing = "fiml", estimator = "MLR")
summary(fit_pars, fit.measures = TRUE, standardized = TRUE)
```
